# Bis[*N*,*N*-bis­(2-hydroxy­ethyl)dithio­carbamato-κ^2^ *S*,*S*′]copper(II)

**DOI:** 10.1107/S160053680904999X

**Published:** 2009-11-28

**Authors:** Lan-Feng Hou, Yun Zhong, Yan Mei, Jun Fan

**Affiliations:** a School of Basical Science, East China Jiaotong University, Nanchang 330013, People’s Republic of China; b School of Chemistry and Environment, South China Normal University, Guangzhou 510006, People’s Republic of China

## Abstract

In the title compound, [Cu(C_5_H_10_NO_2_S_2_)_2_], the Cu^II^ cation is chelated by two bis­(2-hydroxy­ethyl)dithio­carbamate anions with a distorted square-planar coordination geometry. Inter­molecular O—H⋯O hydrogen bonding is observed between the terminal hydr­oxy groups in the crystal structure.

## Related literature

For the different oxidation state of Cu in copper–dithio­carbamate complexes, see: Cardell *et al.* (2006 ▶); Zhang *et al.* (2004 ▶); Jian *et al.* (1999 ▶); Hogarth *et al.* (2000 ▶). For the Cu—S bond distances in a related structure, see: Jian *et al.* (2003 ▶).
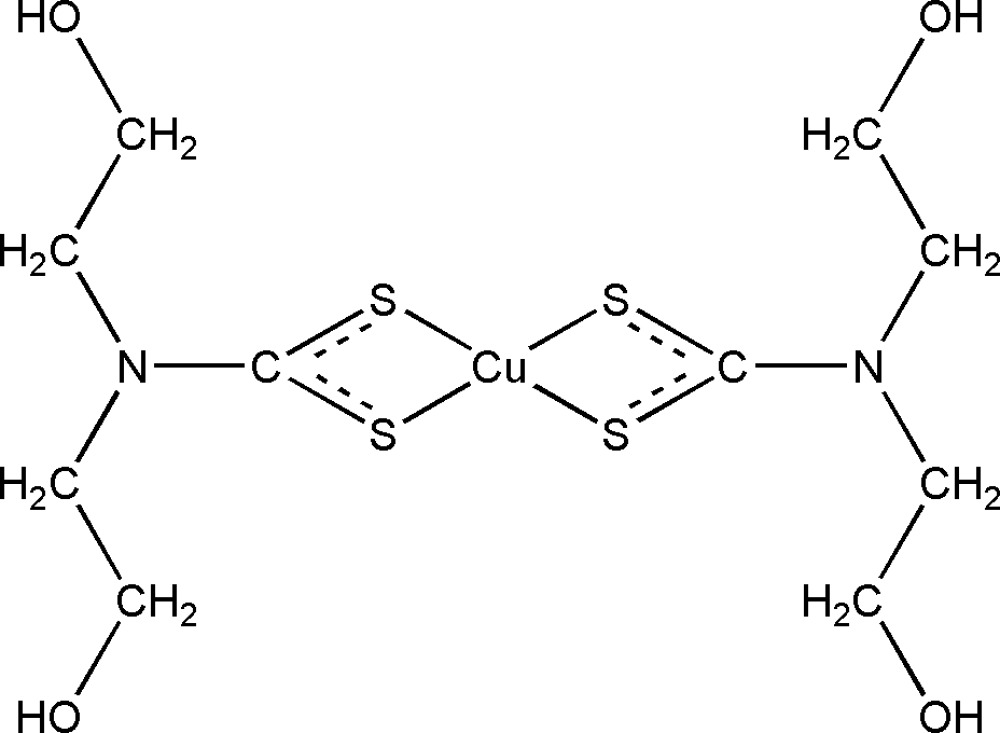


## Experimental

### 

#### Crystal data

[Cu(C_5_H_10_NO_2_S_2_)_2_]
*M* *_r_* = 424.06
Monoclinic, 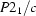

*a* = 11.1088 (9) Å
*b* = 14.7047 (11) Å
*c* = 11.3401 (9) Å
β = 118.253 (1)°
*V* = 1631.7 (2) Å^3^
*Z* = 4
Mo *K*α radiation
μ = 1.86 mm^−1^
*T* = 298 K
0.35 × 0.35 × 0.30 mm

#### Data collection

Bruker SMART CCD area-detector diffractometer
Absorption correction: multi-scan (*SADABS*; Sheldrick, 1996 ▶) *T*_min_ = 0.562, *T*_max_ = 0.605
8158 measured reflections
2928 independent reflections
2652 reflections with *I* > 2σ(*I*)
*R*_int_ = 0.068

#### Refinement

*R*[*F* ^2^ > 2σ(*F* ^2^)] = 0.035
*wR*(*F* ^2^) = 0.095
*S* = 1.03
2928 reflections
190 parameters
H-atom parameters constrained
Δρ_max_ = 1.74 e Å^−3^
Δρ_min_ = −0.56 e Å^−3^

### 

Data collection: *SMART* (Bruker, 1998 ▶); cell refinement: *SAINT* (Bruker, 1999 ▶); data reduction: *SAINT* program(s) used to solve structure: *SHELXS97* (Sheldrick, 2008 ▶); program(s) used to refine structure: *SHELXL97* (Sheldrick, 2008 ▶); molecular graphics: *ORTEP-3 for Windows* (Farrugia, 1997 ▶); software used to prepare material for publication: *SHELXL97*.

## Supplementary Material

Crystal structure: contains datablocks I, global. DOI: 10.1107/S160053680904999X/xu2681sup1.cif

Structure factors: contains datablocks I. DOI: 10.1107/S160053680904999X/xu2681Isup2.hkl

Additional supplementary materials:  crystallographic information; 3D view; checkCIF report

## Figures and Tables

**Table 1:** Selected bond lengths (Å)

Cu1—S1	2.3026 (8)
Cu1—S2	2.3201 (8)
Cu1—S3	2.3148 (8)
Cu1—S4	2.2999 (8)

**Table 2:** Hydrogen-bond geometry (Å, °)

*D*—H⋯*A*	*D*—H	H⋯*A*	*D*⋯*A*	*D*—H⋯*A*
O1—H1⋯O4^i^	0.82	1.86	2.654 (4)	162
O2—H2⋯O1^ii^	0.82	2.29	2.694 (3)	111
O3—H3⋯O2^iii^	0.82	1.94	2.744 (3)	166
O4—H4⋯O3^iv^	0.82	1.90	2.677 (3)	157

Symmetry codes: (i) 
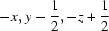
; (ii) 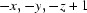; (iii) 
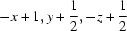
; (iv) 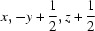.

**Fig. 1.**
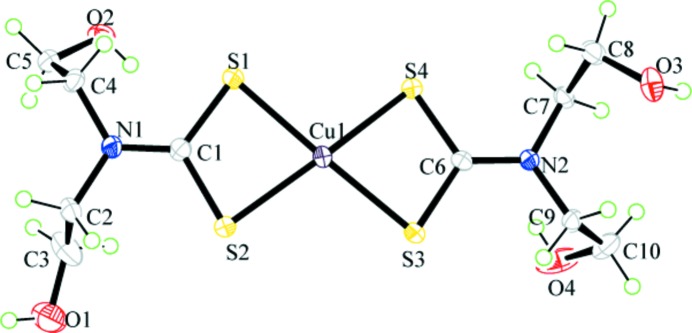
The molecular structure of the title compound with 50% probability displacement ellipsoids for non-H atoms.

## supplementary crystallographic information

### Comment

Metal dithiocarbamate complexes have been extensively studied. Monomeric, dimeric, polymeric, two-dimensional and three-dimensional structures are all featured amongst these complexes. In copper dithiocarbamate complexes, the copper oxidation states I–III have been accessible (Cardell *et al.*, 2006; Zhang *et al.*, 2004; Jian *et al.*, 1999; Hogarth *et al.*, 2000) because dithiocarbamates have capability to stabilize transition metals in a wide range of oxidation states. The title complex (I), was synthesized and characterized by X-ray crystal structure analysis.

The title complex has a monomeric structure (shown as Fig.1). The copper atom, which has distorted square-planar geometry, was coordinated to four sulfur atoms of two 2-hydroxyethyldithiocarbamate ligands. The Cu—S distances ranged from 2.2999 (8) to 2.3201 (8) Å. This is consistent with the literature precedents (Jian, 2003). The interesting feature of this structure is the presence of hydrogen bonding between molecules, owing to the presence of hydrogen-bonding functionality in the N-bound residues. The structure can be thought of as being comprised of layers held together primarily by O—H–O interactions in the *bc* plane; see Table 2 for geometric parameters describing the hydrogen-bonding interactions. Successive layers stack parallel to the a direction, held together by O—H–O interactions.

### Experimental

NaOH(0.04 g, 1.0 mmol), NH(CH_2_CH_2_OH)_2_ (0.105 g, 1.0 mmol) and CS_2_ (0.092 g, 1.2 mmol) was stirred for 1 h in methanol (25 ml) at room temperature, to this solution, CuCl_2_ (0.067 g, 0.5 mmol) was added. The mixture was stirred for 2 h at room temperature and the precipitate was filtered off. Black crystals were obtained from the slow evaporation of the filtrate.

### Refinement

H atoms were placed in calculated positions with C—H = 0.97 and O—H = 0.82 Å, and refined in riding mode with *U*_iso_(H) = 1.2*U*_eq_(C) and 1.5*U*_eq_(O). The highest peak in the final difference Fourier map is 1.1 Å apart from the C1 atom.

### Crystal data

**Table:**

[Cu(C_5_H_10_NO_2_S_2_)_2_]	*F*(000) = 876
*M**_r_* = 424.06	*D*_x_ = 1.726 Mg m^−^^3^
Monoclinic, *P*2_1_/*c*	Mo *K*α radiation, λ = 0.71073 Å
Hall symbol: -P 2ybc	Cell parameters from 5620 reflections
*a* = 11.1088 (9) Å	θ = 2.5–28.1°
*b* = 14.7047 (11) Å	µ = 1.86 mm^−^^1^
*c* = 11.3401 (9) Å	*T* = 298 K
β = 118.253 (1)°	Prism, black
*V* = 1631.7 (2) Å^3^	0.35 × 0.35 × 0.30 mm
*Z* = 4	

### Data collection

**Table:**

Bruker SMART CCD area-detector diffractometer	2928 independent reflections
Radiation source: fine-focus sealed tube	2652 reflections with *I* > 2σ(*I*)
graphite	*R*_int_ = 0.068
φ and ω scans	θ_max_ = 25.3°, θ_min_ = 2.1°
Absorption correction: multi-scan (*SADABS*; Sheldrick, 1996)	*h* = −13→9
*T*_min_ = 0.562, *T*_max_ = 0.605	*k* = −16→17
8158 measured reflections	*l* = −7→13

### Refinement

**Table:**

Refinement on *F*^2^	Primary atom site location: structure-invariant direct methods
Least-squares matrix: full	Secondary atom site location: difference Fourier map
*R*[*F*^2^ > 2σ(*F*^2^)] = 0.035	Hydrogen site location: inferred from neighbouring sites
*wR*(*F*^2^) = 0.095	H-atom parameters constrained
*S* = 1.03	*w* = 1/[σ^2^(*F*_o_^2^) + (0.0446*P*)^2^ + 1.7228*P*] where *P* = (*F*_o_^2^ + 2*F*_c_^2^)/3
2928 reflections	(Δ/σ)_max_ < 0.001
190 parameters	Δρ_max_ = 1.74 e Å^−^^3^
0 restraints	Δρ_min_ = −0.56 e Å^−^^3^

### Special details

**Table:**

Geometry. All e.s.d.'s (except the e.s.d. in the dihedral angle between two l.s. planes) are estimated using the full covariance matrix. The cell e.s.d.'s are taken into account individually in the estimation of e.s.d.'s in distances, angles and torsion angles; correlations between e.s.d.'s in cell parameters are only used when they are defined by crystal symmetry. An approximate (isotropic) treatment of cell e.s.d.'s is used for estimating e.s.d.'s involving l.s. planes.
Refinement. Refinement of *F*^2^ against ALL reflections. The weighted *R*-factor *wR* and goodness of fit *S* are based on *F*^2^, conventional *R*-factors *R* are based on *F*, with *F* set to zero for negative *F*^2^. The threshold expression of *F*^2^ > σ(*F*^2^) is used only for calculating *R*-factors(gt) *etc*. and is not relevant to the choice of reflections for refinement. *R*-factors based on *F*^2^ are statistically about twice as large as those based on *F*, and *R*- factors based on ALL data will be even larger.

### Fractional atomic coordinates and isotropic or equivalent isotropic displacement parameters (Å^2^)

**Table:**

	*x*	*y*	*z*	*U*_iso_*/*U*_eq_	
C1	0.0521 (3)	−0.05853 (18)	0.2261 (3)	0.0192 (6)	
C2	−0.1504 (3)	−0.0869 (2)	0.2496 (3)	0.0233 (6)	
H2A	−0.1983	−0.1411	0.2531	0.028*	
H2B	−0.2019	−0.0601	0.1614	0.028*	
C3	−0.1424 (4)	−0.0196 (3)	0.3548 (4)	0.0406 (8)	
H3A	−0.1207	−0.0520	0.4371	0.049*	
H3B	−0.0697	0.0236	0.3735	0.049*	
C4	0.0381 (3)	−0.20342 (18)	0.3257 (3)	0.0212 (6)	
H4A	0.1013	−0.2229	0.2942	0.025*	
H4B	−0.0383	−0.2454	0.2901	0.025*	
C5	0.1091 (3)	−0.2093 (2)	0.4765 (3)	0.0227 (6)	
H5A	0.0560	−0.1763	0.5100	0.027*	
H5B	0.1130	−0.2725	0.5028	0.027*	
C6	0.3436 (3)	0.17401 (18)	0.0766 (2)	0.0162 (5)	
C7	0.5611 (3)	0.20374 (19)	0.0815 (3)	0.0190 (6)	
H7A	0.5985	0.1702	0.1650	0.023*	
H7B	0.6123	0.2600	0.0980	0.023*	
C8	0.5805 (3)	0.1480 (2)	−0.0214 (3)	0.0255 (6)	
H8A	0.6740	0.1264	0.0185	0.031*	
H8B	0.5209	0.0953	−0.0462	0.031*	
C9	0.3588 (3)	0.30637 (19)	−0.0457 (3)	0.0206 (6)	
H9A	0.2618	0.3096	−0.0736	0.025*	
H9B	0.3693	0.3001	−0.1255	0.025*	
C10	0.4260 (3)	0.3936 (2)	0.0247 (3)	0.0307 (7)	
H10A	0.5215	0.3927	0.0462	0.037*	
H10B	0.3827	0.4445	−0.0347	0.037*	
Cu1	0.18494 (3)	0.05408 (2)	0.13092 (3)	0.02051 (13)	
N1	−0.0131 (2)	−0.11176 (16)	0.2714 (2)	0.0194 (5)	
N2	0.4167 (2)	0.22570 (16)	0.0394 (2)	0.0166 (5)	
O1	−0.2655 (3)	0.02700 (18)	0.3122 (3)	0.0447 (6)	
H1	−0.3242	−0.0082	0.3092	0.067*	
O2	0.2441 (2)	−0.17337 (15)	0.5363 (2)	0.0270 (5)	
H2	0.2414	−0.1197	0.5156	0.040*	
O3	0.5511 (2)	0.19925 (15)	−0.13961 (19)	0.0290 (5)	
H3	0.6027	0.2431	−0.1195	0.044*	
O4	0.4164 (3)	0.40549 (16)	0.1437 (2)	0.0457 (7)	
H4	0.4534	0.3626	0.1944	0.069*	
S1	0.20586 (7)	−0.08469 (5)	0.23294 (7)	0.02249 (18)	
S2	−0.01496 (8)	0.04398 (5)	0.14746 (8)	0.02730 (19)	
S3	0.17688 (7)	0.19631 (5)	0.03995 (7)	0.01979 (17)	
S4	0.40396 (7)	0.07346 (5)	0.16331 (7)	0.02160 (18)	

### Atomic displacement parameters (Å^2^)

**Table:**

	*U*^11^	*U*^22^	*U*^33^	*U*^12^	*U*^13^	*U*^23^
C1	0.0173 (13)	0.0192 (14)	0.0184 (13)	−0.0016 (11)	0.0064 (11)	−0.0002 (10)
C2	0.0194 (14)	0.0258 (15)	0.0272 (15)	−0.0037 (12)	0.0130 (12)	−0.0013 (12)
C3	0.0315 (18)	0.051 (2)	0.047 (2)	−0.0082 (17)	0.0249 (16)	−0.0178 (18)
C4	0.0210 (14)	0.0159 (13)	0.0253 (14)	−0.0022 (11)	0.0097 (12)	0.0012 (11)
C5	0.0208 (14)	0.0214 (14)	0.0258 (15)	0.0013 (12)	0.0110 (12)	0.0039 (11)
C6	0.0163 (12)	0.0175 (13)	0.0151 (12)	0.0001 (11)	0.0076 (10)	−0.0040 (10)
C7	0.0144 (13)	0.0229 (14)	0.0202 (13)	−0.0002 (11)	0.0086 (11)	0.0004 (11)
C8	0.0250 (15)	0.0253 (15)	0.0311 (15)	0.0012 (13)	0.0173 (13)	−0.0021 (12)
C9	0.0172 (13)	0.0227 (14)	0.0203 (13)	0.0003 (11)	0.0077 (11)	0.0057 (11)
C10	0.0266 (16)	0.0206 (15)	0.0334 (17)	−0.0023 (13)	0.0047 (13)	0.0066 (13)
Cu1	0.0192 (2)	0.0191 (2)	0.0275 (2)	0.00087 (14)	0.01459 (16)	0.00434 (13)
N1	0.0161 (11)	0.0186 (11)	0.0226 (12)	−0.0017 (10)	0.0084 (9)	0.0018 (9)
N2	0.0146 (11)	0.0173 (11)	0.0182 (11)	−0.0005 (9)	0.0079 (9)	0.0009 (9)
O1	0.0341 (13)	0.0424 (14)	0.0581 (16)	0.0015 (12)	0.0223 (12)	−0.0075 (12)
O2	0.0191 (10)	0.0286 (11)	0.0287 (11)	0.0026 (9)	0.0076 (8)	0.0000 (9)
O3	0.0321 (12)	0.0350 (12)	0.0256 (10)	−0.0108 (10)	0.0183 (9)	−0.0059 (9)
O4	0.0601 (17)	0.0282 (12)	0.0299 (12)	0.0181 (12)	0.0058 (11)	−0.0052 (10)
S1	0.0161 (3)	0.0221 (4)	0.0296 (4)	0.0025 (3)	0.0111 (3)	0.0073 (3)
S2	0.0251 (4)	0.0220 (4)	0.0423 (4)	0.0070 (3)	0.0221 (3)	0.0120 (3)
S3	0.0157 (3)	0.0196 (3)	0.0263 (4)	0.0013 (3)	0.0118 (3)	0.0025 (3)
S4	0.0195 (3)	0.0189 (3)	0.0294 (4)	0.0034 (3)	0.0140 (3)	0.0062 (3)

### Geometric parameters (Å, °)

**Table:**

C1—N1	1.324 (4)	C7—C8	1.522 (4)
C1—S1	1.717 (3)	C7—H7A	0.9700
C1—S2	1.730 (3)	C7—H7B	0.9700
C2—N1	1.472 (4)	C8—O3	1.434 (4)
C2—C3	1.519 (4)	C8—H8A	0.9700
C2—H2A	0.9700	C8—H8B	0.9700
C2—H2B	0.9700	C9—N2	1.471 (3)
C3—O1	1.396 (4)	C9—C10	1.508 (4)
C3—H3A	0.9700	C9—H9A	0.9700
C3—H3B	0.9700	C9—H9B	0.9700
C4—N1	1.479 (3)	C10—O4	1.414 (4)
C4—C5	1.510 (4)	C10—H10A	0.9700
C4—H4A	0.9700	C10—H10B	0.9700
C4—H4B	0.9700	Cu1—S1	2.3026 (8)
C5—O2	1.423 (3)	Cu1—S2	2.3201 (8)
C5—H5A	0.9700	Cu1—S3	2.3148 (8)
C5—H5B	0.9700	Cu1—S4	2.2999 (8)
C6—N2	1.319 (4)	O1—H1	0.8200
C6—S4	1.726 (3)	O2—H2	0.8200
C6—S3	1.727 (3)	O3—H3	0.8200
C7—N2	1.477 (3)	O4—H4	0.8200
			
N1—C1—S1	124.4 (2)	O3—C8—H8A	109.1
N1—C1—S2	122.3 (2)	C7—C8—H8A	109.1
S1—C1—S2	113.23 (16)	O3—C8—H8B	109.1
N1—C2—C3	111.0 (2)	C7—C8—H8B	109.1
N1—C2—H2A	109.4	H8A—C8—H8B	107.8
C3—C2—H2A	109.4	N2—C9—C10	112.7 (2)
N1—C2—H2B	109.4	N2—C9—H9A	109.1
C3—C2—H2B	109.4	C10—C9—H9A	109.1
H2A—C2—H2B	108.0	N2—C9—H9B	109.1
O1—C3—C2	111.3 (3)	C10—C9—H9B	109.1
O1—C3—H3A	109.4	H9A—C9—H9B	107.8
C2—C3—H3A	109.4	O4—C10—C9	111.6 (3)
O1—C3—H3B	109.4	O4—C10—H10A	109.3
C2—C3—H3B	109.4	C9—C10—H10A	109.3
H3A—C3—H3B	108.0	O4—C10—H10B	109.3
N1—C4—C5	114.6 (2)	C9—C10—H10B	109.3
N1—C4—H4A	108.6	H10A—C10—H10B	108.0
C5—C4—H4A	108.6	S4—Cu1—S1	100.48 (3)
N1—C4—H4B	108.6	S4—Cu1—S3	76.94 (3)
C5—C4—H4B	108.6	S1—Cu1—S3	176.35 (3)
H4A—C4—H4B	107.6	S4—Cu1—S2	167.33 (3)
O2—C5—C4	112.7 (2)	S1—Cu1—S2	77.02 (3)
O2—C5—H5A	109.1	S3—Cu1—S2	104.91 (3)
C4—C5—H5A	109.1	C1—N1—C2	120.1 (2)
O2—C5—H5B	109.1	C1—N1—C4	121.8 (2)
C4—C5—H5B	109.1	C2—N1—C4	117.4 (2)
H5A—C5—H5B	107.8	C6—N2—C9	122.0 (2)
N2—C6—S4	123.0 (2)	C6—N2—C7	120.7 (2)
N2—C6—S3	124.5 (2)	C9—N2—C7	117.3 (2)
S4—C6—S3	112.51 (15)	C3—O1—H1	109.5
N2—C7—C8	113.3 (2)	C5—O2—H2	109.5
N2—C7—H7A	108.9	C8—O3—H3	109.5
C8—C7—H7A	108.9	C10—O4—H4	109.5
N2—C7—H7B	108.9	C1—S1—Cu1	85.25 (10)
C8—C7—H7B	108.9	C1—S2—Cu1	84.42 (10)
H7A—C7—H7B	107.7	C6—S3—Cu1	84.86 (9)
O3—C8—C7	112.5 (2)	C6—S4—Cu1	85.35 (9)

### Hydrogen-bond geometry (Å, °)

**Table:**

*D*—H···*A*	*D*—H	H···*A*	*D*···*A*	*D*—H···*A*
O1—H1···O4^i^	0.82	1.86	2.654 (4)	162
O2—H2···O1^ii^	0.82	2.29	2.694 (3)	111
O3—H3···O2^iii^	0.82	1.94	2.744 (3)	166
O4—H4···O3^iv^	0.82	1.90	2.677 (3)	157

Symmetry codes: (i) −*x*, *y*−1/2, −*z*+1/2; (ii) −*x*, −*y*, −*z*+1; (iii) −*x*+1, *y*+1/2, −*z*+1/2; (iv) *x*, −*y*+1/2, *z*+1/2.
